# The Precise Detection of HER-2 Expression in Breast Cancer Cell via Au_25_ Probes

**DOI:** 10.3390/nano12060923

**Published:** 2022-03-11

**Authors:** Xu Han, Zhesheng He, Wenchao Niu, Chunyu Zhang, Zhongying Du, Xueyun Gao, Gengmei Xing

**Affiliations:** 1 CAS Key Laboratory for Biomedical Effects of Nanomaterials and Nanosafety, Institute of High Energy Physics, Chinese Academy of Sciences, Beijing 100049, China; hanxu@ihep.ac.cn (X.H.); hezs@ihep.ac.cn (Z.H.); 2 School of Chemical Sciences, University of Chinese Academy of Sciences, Beijing 100049, China; 3 Department of Chemistry and Biology, Beijing University of Technology, Beijing 100124, China; niuwc@ihep.ac.cn (W.N.); zhangchunyu@sdfmu.edu.cn (C.Z.); duzhongying123@foxmail.com (Z.D.)

**Keywords:** triple-negative breast cancer, Au_25_ probes, HER-2, ICP-MS

## Abstract

Triple-negative breast cancer (TNBC) accounts for nearly one-quarter of all breast cancer cases, but effective targeted therapies for this disease remain elusive because TNBC cells lack the expression of the most common three receptors seen in other subtypes of breast cancers. The medium-term diagnosis of breast cancers is essential for development and prognosis. According to reports, patients with TNBC may be converted to a positive epidermal growth factor receptor 2(HER-2) after chemotherapy, and trastuzumab treatment will have a better prognosis. Therefore, it is important to accurately quantify the expression of HER-2 in breast cancer cells. Herein, we design a red fluorescent Au_25_ probe synthesized with BSA-biotin as the ligand, which is accurately quantified by HER-2 primary antibody-biotin using the avidin system. The quantitative detection of the expression of HER-2 in breast cancers is helpful for the companion diagnostic of breast cancer treatment and provides follow-up treatment.

## 1. Introduction

TNBC is characterized by tumors that do not express estrogen receptors (ER), progesterone receptors (PR) or HER-2. Despite major advances in understanding the occurrence and treatment of cancers, TNBC remains an aggressive disease that accounts for 15–25% of all breast cancer cases [1,2]. Until now, there is still a lack of effective treatment strategies, due to the lack of ER/PR/HER-2 expression, making TNBC unsusceptible to current targeted therapies, thus resulting in a high mortality and recurrence rate.

An effective treatment strategy is to make TNBC express one or several markers of the ER/PR/HER-2 during the treatment process, and then we can select targeted drugs for treatment in the companion diagnosis process. A companion diagnostic is a test for a specific biomarker approved by the United States Food and Drug Administration (FDA), expecting patients to get a specific and associated therapy. In the past decade, as people’s interest in precision medicine has been growing, the principle of companion diagnostics has gained more and more attention among laboratory professionals, clinicians and even patients. Chemotherapeutic drugs such as paclitaxel are commonly used in the clinical treatment of TNBC, but studies have shown that the use of these chemotherapeutic drugs has a low pathological complete remission (PCR) rate of only 25.9% [3]. Kasami et al. found that HER-2 changed from negative to over-expressed in some patients during breast cancer chemotherapy [4]. Trastuzumab has been shown to be effective for patients with breast cancers of an over-expressed HER-2 [5,6]. Therefore, we propose a new treatment strategy, which is to detect the expression of ER/PR/HER-2 during treatment with chemotherapeutic drugs, and then select targeted antibody drugs based on protein expression.

One of the key issues of the companion diagnostics to TNBC is the accurate quantification of cellular membrane proteins. To date, a series of methods have been used to analyze the expression level of cellular proteins, such as immunoblotting [7], flow cytometry [8] and enzyme-linked immunosorbent assay (ELISA) [9].

Metal clusters prepared with peptide or protein exhibited an ultra-small size (<2 nm), a high photostability, good biocompatibility, an accurate chemical formula and a specific protein-targeting capacity, which were ideal bioanalytical tools attractive to researchers [10,11,12]. In recent years, metal clusters with a precise chemical structure and targeting ability have been developed to quantitatively analyze various types of tumor cell proteins.

Zhang et al. quantified the MT1-MMP and αvβ3 invasion protein by designing functionalized small peptide gold and silver clusters. After demonstrating their ability to quantify proteins in single cells, Au clusters were also used to quantify the expression level of MT1-MMP in primary tumor tissues [13]. However, the antigen targeting of small peptides is not as good as that of primary antibodies, thus a new strategy is to attach primary antibodies to metal clusters. Au_25_ clusters prepared using bovine serum albumin (BSA) have been widely used in bio-imaging analyses and tumor treatment research [14]. Here, BSA coupled with biotin was designed for the synthesis of BSA-Biotin-Au_25_. Due to the strong affinity of biotin and avidin [15,16], avidin can be used as an intermediate to combine BSA-biotin-Au with primary HER-2 antibody-biotin. The BSA-biotin-Au clusters are trifunctional probes with the following functions: (1) specific targeting of HER-2 protein through the primary antibodies on the biotin; (2) in situ fluorescent labeling of HER-2 protein on cells; and (3) precise quantification of HER-2 protein on TNBC cells during the companion diagnostics. For this study, the SK-Br-3(SK) and MDA-MB-231 cell lines of human breast cancers with different expressions of HER-2 were chosen as model systems. We used a BSA-Biotin-Au cluster to achieve specific labeling of the breast cancer cells. Meanwhile, inductively coupled plasma mass spectrometry (ICP-MS) was employed to count Au on these cells, so as to quantify the expression level of HER-2 protein. On the other hand, for triple negative breast cancer MDA-MB-231 cells, we first used chemotherapy drugs paclitaxel of different concentrations to stimulate. Then, trastuzumab was added to these cancer cells, and the results showed that the toxicity of the drug was greatly improved. We believe that the strategy of stimulating TNBC cells to express HER-2 protein through chemotherapeutics and then using antibody drug trastuzumab therapy will help to improve the current dilemma of TNBC treatment.

## 2. Experimental Procedure

### 2.1. Materials

BSA-biotin was synthesized using a solid-phase method (China Shanghai Peptides Co., Ltd., Purity: 98%). The HAuCl_4_·3H_2_O was purchased from Tianjin Fuchen Chemical Reagents Co., China. MDA-MB-231 and SK-BR-3 cell lines of human breast cancers were purchased from Cancer Institute and Hospital, Chinese Academy of Medical Sciences. Cell culture medium RPMI 1640, L-15 medium and phosphate-buffered solution were purchased from Gibco. Cell lysis buffer for Western and IP, DAPI staining solution, CCK-8, anti-rabbit lgG-HRP and BCA protein assay kit were obtained from Beyotime Institute of Biotechnology, China. Human HER-2 antibody, HER-2 biotinylated antibody, Avidin and trastuzumab were obtained from Abcam. Paclitaxel was obtained from Sigma. Ultrapure water (18 MΩ) was used in the experiments. All reagents were used as received without modification.

### 2.2. Synthesis of BSA-Biotin-Au_25_ Clusters

A modified method of Xie^’^s was adopted to synthesize Au clusters with BSA-biotin. Aqueous HAuCl_4_ solution (5 mL, 10 mM) was mixed with BSA-biotin solution (5 mL, 50 mg/mL) under vigorous stirring at 37 °C. NaOH solution (0.5 mL, 1 M) was introduced 2 min later, and the reaction was allowed to proceed under vigorous stirring at 37 °C for 12 h. The resultant solution was stored at 4 °C in refrigerator until use.

### 2.3. Characterization of BSA-Biotin-Au_25_ Clusters

The UV-Vis absorption spectrum of BSA-biotin-Au_25_ was measured using a spectrophotometer (Shimadzu uv-1800, Kyoto, Japan). The fluorescence spectrum of BSA-biotin-Au_25_ was obtained with a fluorescence spectrophotometer (Shimadzu rf-5301, Kyoto, Japan). The molecular weight of BSA and BSA-biotin was determined by matrix-assisted laser desorption/ionization time of flight mass spectrometry (MALDI-TOF-MS, Ultraflextreme, Bruker, Breme, Germany).

### 2.4. Fluorescence Imaging of SK Cells through HER-2 Primary Antibody-Biotin Avidin and BSA-Biotin-Fitc

SK cells fixed with 4% of paraformaldehyde were incubated with HER-2 primary antibody-biotin at room temperature for 1 h, which were then incubated with avidin for 45 min and finally with BSA-biotin-FITC for 30 min/45 min/60 min, respectively. The cells were then washed with PBS and observed using a confocal laser scanning microscope (CLSM, UltraVIEW vox+ Nikon, PerkinElmer, Waltham, MA, USA).

### 2.5. Fluorescence Imaging of SK Cells through HER-2 Primary Antibody-Biotin Avidin and BSA-Biotin-Au_25_

SK cells were fixed with 4% of paraformaldehyde and incubated with HER-2 primary antibody-biotin for 1 h at room temperature, which were then incubated with avidin for 45 min, and finally with BSA-biotin-Au_25_ of different concentrations (1.5–50 μM) for 45 min. The cells were then washed with PBS and observed using CLSM.

### 2.6. Expression Level of HER-2 in MDA-MB-231 Cells via Western Blotting

The 1 × 10^5^ cells/mL MDA-MB-231 cells were seeded in 6-well plates for 12 h in the control group using a L-15 medium for 24 h and with L-15 medium supplemented by 0.5 ug/mL paclitaxel in the experimental group. The whole cell protein was collected, the cells were washed with PBS and lysed with ripa buffer containing protease inhibitors at 4 °C for 15 min. After centrifugation at 12,000× *g* for 5 min, the supernatant was collected. Then, 10% SDS polyacrylamide gel was used to separate the same protein, which was then transferred to the PVDF membrane. Seal with 0.3% BSA solution for 1 h at room temperature, then mix with HER-2, and β-tublin antibodies were incubated at 4 °C overnight. Then, membranes were washed with TBST buffer and incubated with secondary antibodies at room temperature for 2 h. The results were observed using Amersham ECLTM Prime Western Blotting Detection Reagent (GE Healthcare, Amersham, UK).

### 2.7. Fluorescence Imaging of MDA-MB-231 Cells through HER-2 Primary Antibody-Biotin Avidin and BSA-Biotin-Au_25_ before and after Paclitaxel Treatment

One group of MDA-MB-231 cells were fixed with 4% paraformaldehyde and incubated with HER-2 primary antibody-biotin for 1 h at room temperature, which were then incubated with avidin for 45 min, and finally with 12 μM of BSA-biotin-Au_25_ for 30/45/60 min. Another group of MDA-MB-231 cells were incubated with 0.5 μg/mL paclitaxel and the above steps were repeated.

### 2.8. Cell Viability Test

To test the sensitivity of MDA-MB-231 cells to drugs of different concentrations (0–10 µg/mL) (paclitaxel and trastuzumab), 5 × 10^3^ cells/well MDA-MB-231 cells were inoculated on 96-well plates and cultured for 24 h. Gradient doses of paclitaxel and trastuzumab were incubated with cells for 24 h. Then, the cells were washed three times with PBS and incubated in a medium containing 10% (*v/v*) of the CCK-8 reagent for 30 min at 37 °C. In the next experiment, we repeated a batch of cells, which were stimulated by paclitaxel for 24 h, then the above steps were repeated.

### 2.9. ICP-MS Analysis of the Concentration of Au in the SK and MDA-MB-231 Cells

After fixing SK and MDA-MB-231 cells with 4% paraformaldehyde for 30 min, the cells were first incubated with HER-2 primary antibody-biotin for 1 h, then with avidin for 45 min, and finally with 1.5–100 µM BSA-biotin-Au_25_ for 45 min. The cells were then counted and digested with a mixed solution (V_H2O2_/V_HNO3_ = 1:3). Then, the samples were transferred to the microwave reaction system. Finally, an aqueous solution containing 2% of HNO_3_ was added to 10 mL, which was the final volume. The precision and accuracy of this technique can be evaluated by the measured concentration and relative standard deviation of the indium concentration. Each experiment was repeated three times.

## 3. Results and Discussion

### 3.1. Preparation of BSA-Biotin-Au_25_ Probes

**Figure 1:**
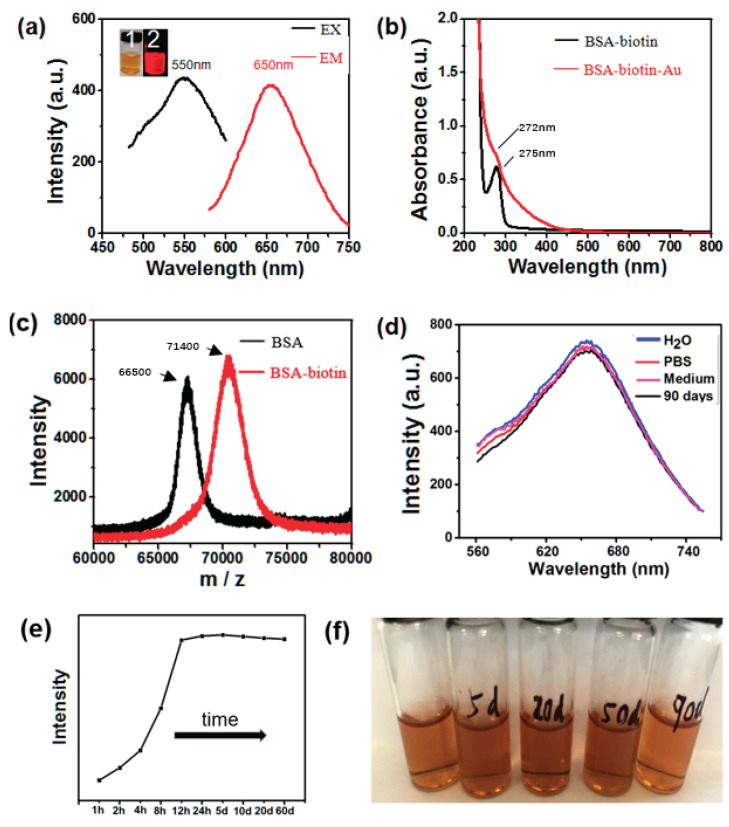
(**a**) The fluorescence excitation (black line, λex = 550 nm), and emission (red line, λem = 650 nm) spectra of the red-emitting BSA-biotin-Au. Digital photos of BSA-biotin-Au under (1) visible and (2) 365 nm portable UV light. (**b**) The UV-vis absorption of BSA-biotin (black line) and BSA-biotin-Au (red line). (**c**) The MALDI-TOF-MS spectra of BSA (66,500 KDa) and BSA-biotin (71,400 KDa). (**d**) The fluorescence intensity of BSA-biotin-Au in different solvents. (**e**) Time-dependent fluorescence intensity curve of BSA-biotin-Au. (**f**) Color change of BSA-biotin-Au aqueous solution at room temperature with different time.

To generate our multifunctional BSA-Biotin-Au_25_ cluster probes, a BSA-biotin template was designed to biomineralize Au clusters (see methods and Figure 1a). To determine whether BSA was successfully linked to biotin, MALDI-TOF-MS was employed. As is shown in Figure 1c, the mass spectrum of BSA peak was at 66,500 *m*/*z*, while that of BSA-biotin peak was at 71,400 *m*/*z*, indicating that BSA was successfully cross-linked to biotin. As is depicted in Figure 1a, BSA-Biotin-Au_25_ clusters exhibited an intense emission peak at 650 nm (red curve, Figure 1a) when excited at 550 nm (black curve, Figure 1a). This result is close to the excitation and emission results of BSA-Au synthesized by Xie, where the clusters also appeared with red fluorescence [14]. In addition, compared with the BSA-biotin absorption peak at 275 nm (black curve, Figure 1b), the absorption spectrum of BSA-Biotin-Au_25_ clusters displayed the characteristic absorption peaks of Au clusters (red curve, 272 nm, Figure 1b). The results are similar to the previously reported absorption peaks of gold clusters, revealing that they have similar optical features [17], indicating the successful construction of BSA-biotin-Au_25_ clusters. Figure 1c,d show the MASS spectra and changes in fluorescence during the formation of BSA-Biotin-Au_25_ clusters and their stability. The fluorescence of the BSA-biotin-Au_25_ clusters was stable at room temperature in the buffered media, and there was no significant change over 90 days (Figure 1e,f). The method to synthesize the probes is benign and the products are of a precise molecular form and a stable ability in aqueous solution, which is therefore beneficial for later studies on cell marking.

### 3.2. The Specific Targeting of BSA-Biotin-Fitc and Primary Antibody with Biotin to Her-2 Protein with Avidin as a Mediator

**Figure 2:**
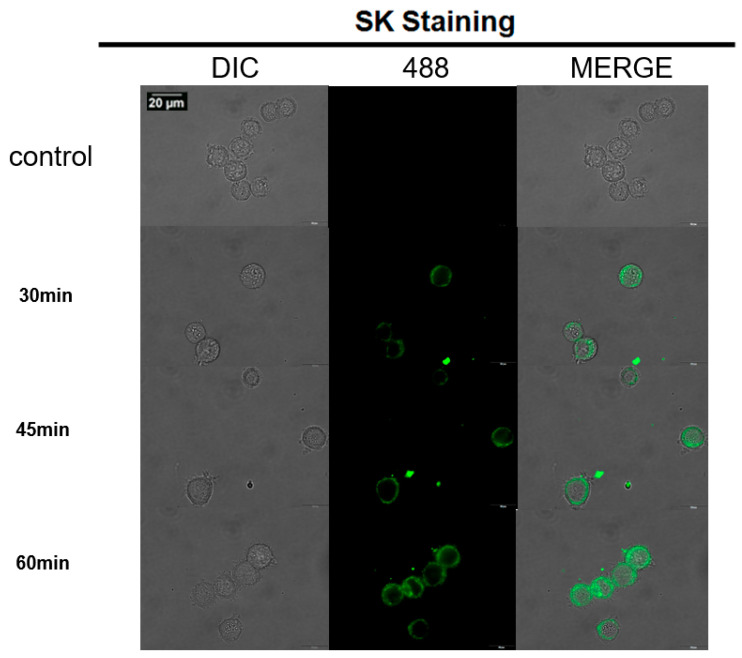
Fluorescence imaging of BSA-biotin-fitc labeled HER-2 in SK cells by avidin and primary antibody-biotin at different time points.

The HER-2 in SK cells is specifically bound by the biotin-linked primary antibodies, then the biotin is connected with avidin in the primary antibodies, and finally BSA-biotin-fitc is connected to avidin. CLSM was employed to evidence the specificity of BSA-biotin-FITC for HER-2 expressed on breast cancer SK cells (Figure 2). From Figure 2, we can see that the fluorescence tends to be saturated after 45 min of incubation. When incubated for 60 min, there was fluorescence infiltrating into the cytoplasm of the cells. The biotin-avidin plays a key role in this cell tagging approach, the specific and strong affinity between biotin and avidin ensures that the BSA-biotin-fitc is well tagged on the over-expressed SK cells in HER-2, and this approach may be further developed for other cell marking.

### 3.3. The Specific Targeting of BSA-Biotin-Au_25_ Clusters and Primary Antibodies Containing Biotin to Her-2 Protein with Avidin as a Mediator

**Figure 3:**
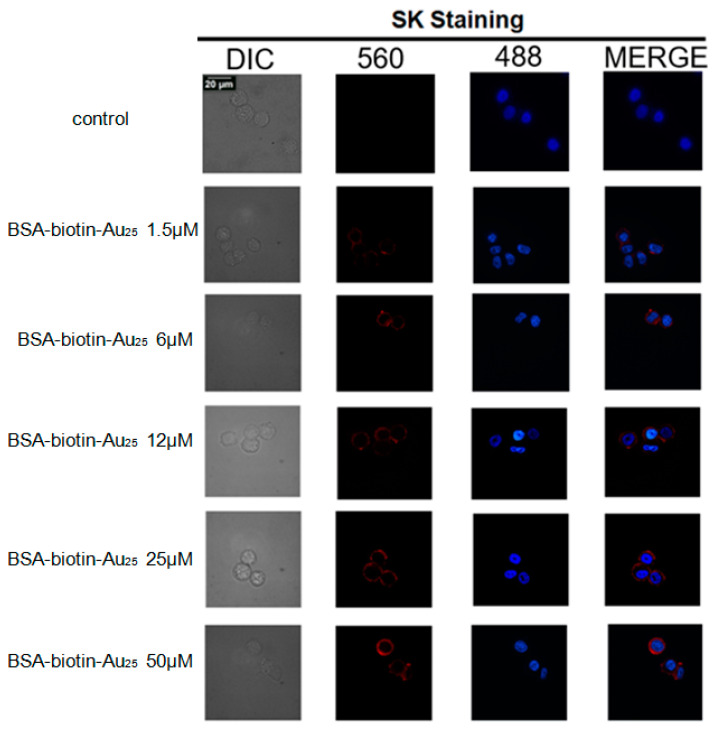
Fluorescence imaging BSA-biotin-Au_25_-labeled SK cells of different concentrations for 45 min.

It has been confirmed that BSA-biotin-fitc can label HER-2 protein in SK cells. Do BSA-biotin gold clusters also have this feature? In order to accurately quantify the protein, we studied the optimal labeling conditions in cell assays, for which we referred to Zhang’s method [18]. As is shown in Figure 3, the red fluorescence represents the intrinsic fluorescence of BSA-biotin-Au_25_, which is located on the cell membrane. As can be seen from the fluorescence intensity (Figure 3), when the concentration of BSA-biotin-Au_25_ ranging from 1.5–50 μM increased, the fluorescence intensity of SK cells increased. When the labeled SK cells were saturated, their fluorescence intensity did not change significantly when the concentration of BSA-biotin-Au_25_ was high. The probe was located at 12 μM, and BSA-biotin-Au_25_ was the best culture condition for SK cells. By applying probes of serial concentration in SK cell culture media, the optimized concentration of probes for marking the HER-2 in SK cells were well sorted, such optimization would ensure the thorough tagging of HER-2 on the surface of SK cells and make sure that the final result would be more accurate.

### 3.4. Changes in the Expression of HER-2 Protein in Triple-Negative Breast Cancer MDA-MB-231 Cells before and after Paclitaxel Treatment

**Figure 4:**
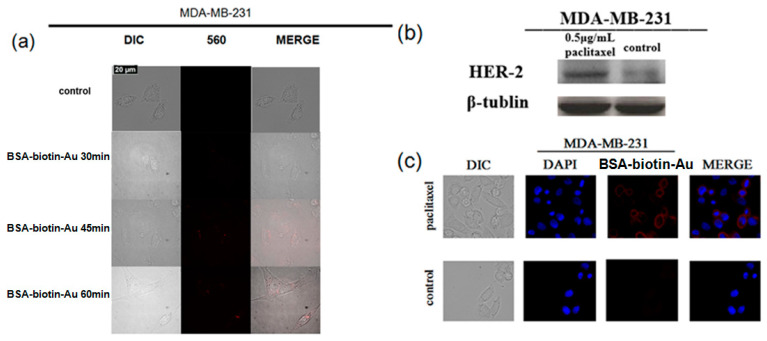
(**a**) Fluorescence imaging of 12 μM BSA-biotin-Au_25_ labeled MDA-MB-231 cells at different time points. (**b**) HER-2 protein expressed in MDA-MB-231 cells before and after paclitaxel stimulation (**c**) Fluorescence imaging of BSA-biotin-Au_25_ labeled MDA-MB-231 cells before and after paclitaxel stimulation.

There is almost no HER-2 protein on the triple-negative breast cancer MDA-MB-231 cell membrane, but its expression increased after paclitaxel treatment. As is shown in Figure 4a, the red fluorescence of BSA-Biotin-Au clusters is almost invisible on the MDA-MB-231 cell membrane, which means that there is almost no HRE-2 protein on it. Only faint bands can be seen in the Western blotting results of MDA-MB-231 cell lines in the control group, which further proves the accuracy of BSA-Biotin-Au clusters to label the HER-2 protein (Figure 4b). However, after a 0.5 μg/mL paclitaxel treatment for 24 h, the expression level of HER-2 protein significantly increased (Figure 4b). The results of CLSM imaging further proved the occurrence in changes in the expression of HER-2 on MDA-MB-231 cell lines before and after paclitaxel treatment. Moreover, the expression of HER-2 on MDA-MB-231 cells was further verified using a BSA-Biotin-Au_25_ cluster probes. As is depicted in Figure 4c, after treatment with paclitaxel, the MDA-MB-231 cells were labeled by BSA-Biotin-Au_25_ clusters and emitted red fluorescence, while there was no fluorescence signal in MDA-MB-231 cells treated with PBS. These results prove that our BSA-Biotin-Au cluster-probe labeling method is as accurate as the commercial antibody immunoblotting method, noting that the immunoblotting process is cumbersome. On the other hand, the expression of the HER-2 protein on triple-negative breast cancer MDA-MB-231 cells increases during treatment with the chemotherapeutic drug paclitaxel, which provides a prerequisite for companion diagnoses.

### 3.5. Monitor the Changes in the Expression of HER-2 Protein on MDA-MB-231 Cells in Companion Diagnostics

**Figure 5:**
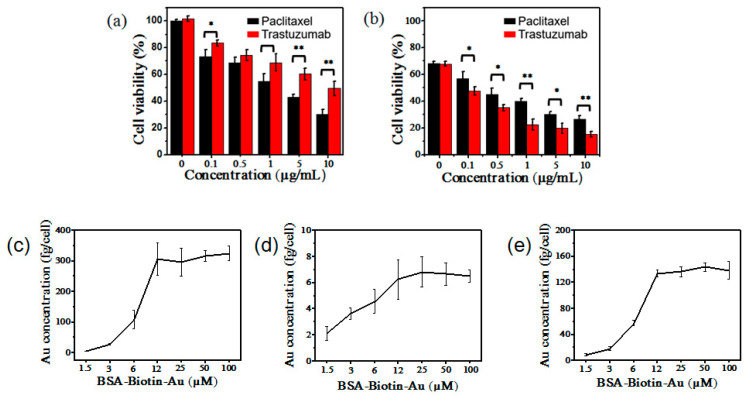
(**a**) Cytotoxicity of paclitaxel and trastuzumab of different concentrations on MDA-MB-231 cells The significant differences between groups were expressed by * *p* < 0.05, ** *p* < 0.01. (**b**) Cytotoxicity of paclitaxel and trastuzumab of different concentrations on MDA-MB-231 cells after the stimulation of 0.5 μg/mL paclitaxel (**c**) ICP-MS of SK cells. (**d**) ICP-MS of MDA-MB-231 cells (**e**) ICP-MS of MDA-MB-231 cells with the stimulation of 0.5 μg/mL paclitaxel.

As is mentioned above, the use of these chemotherapeutic drugs has a lower pathological complete remission for TNBC cancer. Due to the lack of a high expression of HER-2 protein, trastuzumab did not exhibit significant toxicity to MDA-MB-231 cells. The IC50 concentration of paclitaxel and trastuzumab in MDA-MB-231 cells was 1 to 10 μg/mL (Figure 5a). After 0.5 μg/mL treatment of paclitaxel for 24 h, paclitaxel and trastuzumab with different concentrations were introduced into MDA-MB-231 cells for another 24 h. As is depicted in Figure 5b, the IC50 concentration of paclitaxel and trastuzumab in MDA-MB-231 cells was 0.1 to 1 μg/mL. When trastuzumab (1 μg/mL) alone was used to treat MDA-MB-231 cells, the cell survival rate reached 70%, but when trastuzumab (1 μg/mL) was treated after treatment with paclitaxel (0.5 μg/mL for 24 h), the cell survival rate was only 20% (Figure 5a,b). These treatments drastically increased the toxicity of trastuzumab in TNBC MDA-MB-231 cells. For 11 patients whose serum HER-2/*neu* protein level was tested for their ability to predict the response to therapies, it was found in the studies that eight patients (73%) presented an elevated serum HER-2/*neu* protein level, which predicted therapeutic resistance [19,20,21,22,23]. It indicates that the increase in HER-2 is indeed related to the drug resistance of breast cancers. We speculated that in the stimulating process of MDA-MB-231, cells with low-concentration paclitaxel, the expression of HER-2 protein was increased, which provided a target for the follow-up treatment of trastuzumab, thus significantly enhancing the cytotoxicity. To verify this hypothesis, we used BSA-Biotin-Au clusters combined with ICP-MS to quantify changes in the dynamic expression of HER-2 protein on cells. As is shown in Figure 5c, we first performed labeling and quantification of SK cells with highly expressed HER-2 protein. With the increase in the Au concentration from 1.5 to 100 μM, the amount of Au increased (Figure 5c), the count of which, up to 12 μM, was stabilized without a significant increase at relatively higher Au concentrations, indicating that all HER-2 proteins on the SK cell membrane had been labeled and quantified. The average mass of Au on SK cells was 298.84 ± 52.08 fg per cell. Similarly, that of Au on MDA-MB-231 cells was 6.02 ± 1.78 fg per cell (Figure 5d). However, after stimulation with paclitaxel, the HER-2 protein expressed by MDA-MB-231 cells was significantly observed, reaching a concentration of 131.58 ± 5.26 fg per cell (Figure 5e). These results clearly verify why trastuzumab can significantly kill TNBC cells after being stimulated by paclitaxel. For companion diagnostic tests, the guidelines are generally issued to guide pathologists in the interpretation and scoring of the staining. The HER-2 scoring guidelines recommended by ASCO/CAP-classified that HER-2 were expressed as 0 (no staining or faint incomplete membrane staining observed in ≤10% of tumor cells), 1+ (faint/barely perceptible incomplete membrane staining in >10% of tumor cells), 2+ (circumferential membrane staining that is incomplete and/or weak/moderate within >10% of tumor cells or complete and circumferential membrane staining that is intense within ≤10% of tumor cells) or 3+ (circumferential membrane staining that is complete and intense within >10% of tumor cells). Tumors with a score of 0 or 1+ were considered negative; those scored 2+ were considered equivocal and FISH reflex testing was required; and those scored 3+ were considered positive and eligible for trastuzumab [24]. According to these guidelines, our precise detection of HER-2 could offer a new idea for HER-2 scoring. This companion diagnostics for TNBC provides a strategy for reducing drug use, improving drug efficacy and choosing personalized treatment plans. Our probes can be used to detect the expression of HER-2 during drug treatment well and may provide an accompanying diagnostic approach for breast cancer therapies.

## 4. Conclusions

Companion diagnostics for TNBC have gained momentum in recent years, demonstrating clear benefits including not only choosing a personalized treatment plan for patients, but also reducing the use of drugs. While choosing a companion diagnostic strategy for TNBC, the most difficult problem is to accurately quantify the proteins. In this study, we designed and constructed BSA-Biotin-Au clusters that could specifically target HER-2 proteins on the cell surface. With the inherent fluorescence properties and targeting ability of BSA-Biotin-Au clusters, we were able to perform fluorescence imaging and precise quantification of HER-2 proteins on breast cancer cells. After stimulation with low concentration paclitaxel, the expression of HER-2 proteins on TNBC MDA-MB-231 cells increased. When the HER-2 proteins were exposed, treatment with trastuzumab could dramatically increase the cytotoxicity. Moreover, in this process, changes in the expression of HER-2 proteins on MDA-MB-231cells were quantified through BSA-biotin-Au clusters. This cell protein quantification and monitoring method is beneficial for the clinical selection of smarter treatment decisions for TNBC. In addition, if HER-2 primary antibodies are replaced by other primary antibodies, other target proteins can be quantified using our materials, so that they can be used for the quantification of other proteins. In later studies, we will use our probes in the TNBC tissues from animal models, and further investigate a greater range of tumor cells/tissues to verify the application of these probes.

## Data Availability

All data included in this study are available upon request by contact with the corresponding author.
